# *HIF3A* DNA Methylation Is Associated with Childhood Obesity and ALT

**DOI:** 10.1371/journal.pone.0145944

**Published:** 2015-12-30

**Authors:** Shuo Wang, Jieyun Song, Yide Yang, Yining Zhang, Haijun Wang, Jun Ma

**Affiliations:** Institute of Child and Adolescent Health, School of Public Health, Peking University, Beijing, China; New York University School of Medicine, UNITED STATES

## Abstract

Gene polymorphisms associated so far with body mass index (BMI) can explain only 1.18–1.45% of observed variation in BMI. Recent studies suggest that epigenetic modifications, especially DNA methylation, could contribute to explain part of the missing heritability, and two epigenetic genome-wide analysis studies (EWAS) have reported that *Hypoxia Inducible Factor 3 Alpha Subunit (HIF3A)* methylation was associated with BMI or BMI change. We therefore assessed whether the *HIF3A* methylation is associated with obesity and other obesity-related phenotypes in Chinese children. The subjects included 110 severe obese cases aged 7–17y and 110 normal-weight controls matched by age and gender for measurement of blood DNA methylation levels at the *HIF3A* gene locus using the Sequenom’s MassARRAY system. We observed significantly higher methylation levels in obese children than in controls at positions 46801642 and 46801699 in *HIF3A* gene (*P*<0.05), and found positive associations between methylation and alanine aminotransferase (ALT) levels adjusted by gender, age and BMI at the position 46801699 (r = 0.226, *P* = 0.007). These results suggest that *HIF3A* DNA methylation is associated with childhood obesity, and has a BMI-independent association with ALT. The results provide evidence for identifying epigenetic factors of elivated ALT and may be useful for risk assessment and personalized medicine of liver diseases such as non-alcoholic fatty liver disease (NAFLD).

## Introduction

Obesity is a worldwide health problem and has been associated with many medical conditions, such as type 2 diabetes, hypertension, cardiovascular disease, stroke, and cancers [1–3]. The pathogenesis of obesity is complex, involving multiple interactions among behavioral, environmental, and genetic factors [4]. According to heritability studies, inheritance could account for up to 40–70% of the interindividual variability in body weight [5]. Genome-wide association studies (GWAS) have identified 55 genetic loci associated with obesity or body mass index (BMI), but these loci together explain only 1.18–1.45% of observed variation in BMI, which is a small proportion [6].

Previous epigenetic studies have suggested that DNA methylation, the most stable epigenetic marker, could contribute to explain the missing heritability of obesity [7–10]. Through an EWAS study of methylation patterns in peripheral blood DNA with one discovery cohort and two replication cohorts, Dick et al. [9] uncovered a specific association between higher BMI and increased methylation of 3 cytosine-phosphate-guanine dinucleotides (CpG) sites at the first intron of *HIF3A*, but the casual relationship remained unclear. Demerath et al. [11] validated the results through another EWAS study and found that *HIF3A* methylation was associated with BMI change.

In the current study, we analyzed the methylation level of the gene *HIF3A* in a Chinese 7–17y population including 110 cases and 110 controls, at an expanded region covering 3 previously reported CpG sites and 6 new sites. The study aimed to assess whether there were nearby CpG sites with a stronger effect associated with obesity, and whether there were other obesity-related phenotypes associated with *HIF3A* methylation.

## Materials and Methods

### Subjects

This study included 110 severe obese cases aged 7–17 y and 110 normal-weight age- and gender-matched controls. All participants were Chinese Han ethnics chosen from the study Comprehensive Prevention project for Overweight and Obese Adolescents (CPOOA). As described before [12], the CPOOA subjects were recruited from children aged 7–18 y in 5 elementary and middle schools of Haidian District of Beijing, comprising 637 overweight or obese children and 456 normal-weight children. All the obese individuals in the selected schools were recruited with their voluntary participation. The method of cluster sampling was adopted to recruit non-obese subjects from some classes of each grade in the same schools. The ascertainment strategy for the study groups has been previously described in details previously [13, 14]. We choose those with an age- and gender-specific BMI ≥97^th^ percentile as the severely obese cases, and those with BMI between 15th and 85th percentile as normal-weight controls.

The study was approved by the Ethic committee of Peking University Health Science Center. Written informed consent was provided by all participants and, in the case of minors, their parents. Anthropometric measurements, including height, weight, waist circumference and hip circumference were determined according to standard protocols. Fasting venous blood samples were taken for detection of GLU, TC, TG, HDL-C, LDL-C and ALT by using a biochemical autoanalyzer (Hitachi 7060, Tokyo, Japan). Fasting insulin was determined by radio-immunoassay method (Beijing North Institute of Biological Technology, Beijing, China). HOMA-IR was calculated as fasting insulin (μU/ml)×fasting glucose (mmol/l)/22.5.

### DNA methylation analyses

Genomic DNA was extracted from peripheral blood leukocytes by phenol-chloroform extraction method. Sequenom’s MassARRAY system (Sequenom, San Diego, CA) was used to perform quantitative methylation analyses [15]. This system utilizes MALDI-TOF mass spectrometry in combination with RNA base-specific cleavage (MassCLEAVE). PCR primers for the HIF3A gene were designed using Epidesigner online application (http://www.epidesigner.com). Sequences of primers were: 10mer tagged forward primer: 5’-aggaagagagAGGTTTTGGTTTTGGGTTTAATAAG-3’; T7 promoter tagged reverse primer: 5’-cagtaatacgactcactatagggagaaggctTAAAATAACAACCAACCCCAACTAA-3’. Sequenom’s EpiTYPER protocol includes the method and procedure of methylation data measurement [16]. This method has been widely used to conduct DNA methylation measurement and to do the association analyses with different diseases [17–19]. Bisulfite-treated DNA was PCR amplified in 5μL reactions (94°C for 4 min; 45 cycles of 94°C for 20s, 60.9°C for 30 s, and 72°C for 1 min; 72°C for 3 min), treated with Shrimp Alkaline Phosphatase (Sequenom) (37°C for 20 min), heat inactivated (85°C for 5 min), and then in T-cleavage transcription (37°C for 180 min). Transcription cleavage products were desalted with 6 mg/well of Clean-Resin spotted on a 384-element silicon chip (SpectroCHIP, Sequenom). Mass spectra were acquired using a MassARRAY MALDI-TOF MS (Sequenom), and peak detection, signal-to-noise calculations, quantitative CpG site methylation were performed using proprietary EpiTyper software v1.2.1 (Sequenom).

In terms of quality control, a fully-methylated positive control and a 0-methylated negative control were included for each run. Methylation detection was conducted in duplicate for all the samples. After excluding data with more than 30% disparity between duplicate samples, we then calculated the average values as the methylation levels for each individual at each CpG unit. 10 CpG sites in 6 CpG units were obtained initially (CpG1.2, CpG5, CpG6.7.8, CpG11, CpG12 and CpG13.14), but CpG12 was excluded because of its low call rate (71.8%), remaining 5 units with call rates over 80%. There were 3 samples which failed to give a reliable PCR product, and 5 samples were excluded from analysis samples because of low call rates (<80%). Thus, we have analyzed 5 CpG units (9 CpG sites) of *HIF3A* methylation among 107 obese children and 105 controls.

### Statistical analyses

Statistical analyses were performed using SPSS 18.0 (IBM, Chicago, IL, USA). When comparing the general characteristics and methylation levels between the obese children and controls, categorical variables were compared using Pearson’s χ2-test, whereas group differences for continuous variables were compared using Student t-test. When conducting partial correlation analyses, BMI, WHtR, PBF and all metabolic indicators had been Log converted due to the skewed distribution. The criterion for statistical significance was set at *P* <0.05.

A power analysis was conducted to determine the sample size necessary for testing the methylation difference between obese cases and controls in two-sample T-tests. Based on the CpG7 data in *HIF3A* methylation discovery EWAS study (cg16672562, which had the lowest effect on BMI of all the three sites reported) [9], the mean methylation level of healthy controls was expected to be 0.409, and the standard deviation (SD) was expected to be 0.101. The discovery EWAS study estimated by liner regression that every 0 1 increase in CpG7 (cg16672562) methylation level was associated with a 3 6% higher BMI. In the current study, severe obese children were selected as cases and the average BMI of obese children was more than 3.6% higher as compared to non-obese controls, but the effect of methylation could be different considering the difference of ethnicity, age and study design, so we assumed the mean methylation level of obese cases to be 0.05 higher than controls. Based on those assumptions, a sample size of 108 cases and 108 controls was required to obtain 95% power to test the methylation difference between cases and controls.

## Results

### General characteristics of obese children and controls

General characteristics of obese children and controls are summarized in Table 1. There was no difference between the two groups in age (*P* = 0.934) or gender (*P* = 0.946). Overall, we observed significant differences in the obesity-related phenotypes. Obese children had a higher level of fasting glucose (GLU), total cholesterol (TC), low-density lipoprotein cholesterol (LDL-C), triglycerides (TG), homeostasis model assessment of insulin resistance (HOMA-IR), ALT and a lower level of high-density lipoprotein cholesterol (HDL-C) as compared with controls (*P* <0.001).

**Table 1:** General characteristics of the obese children and controls.

	Obese Children(n = 107)	Controls(n = 105)	*P* Value
Age (years)	11.1(2.6)	11.0(2.6)	0.919
Male	55(51.4)	52(49.5)	0.785
BMI (kg/m^2^)	28.4(3.8)	17.1(2.1)	<0.001
WHtR	0.56(0.05)	0.40(0.03)	<0.001
PBF (%)	31.7(4.1)	13.6(6.3)	<0.001
GLU (mmol/L)	5.5(0.5)	5.2(0.4)	<0.001
TC (mmol/L)	4.2(0.7)	3.9(0.3)	<0.001
LDL-C (mmol/L)	2.4(0.6)	1.9(0.4)	<0.001
HDL-C (mmol/L)	1.3(0.3)	1.7(0.2)	<0.001
TG (mmol/L)	1.3(0.6)	0.7(0.2)	<0.001
HOMA-IR	4.0(2.1)	1.8(0.5)	<0.001
ALT (IU/L)	26.5(31.6)	10.4(5.2)	<0.001

Data are mean(SD) or number(%).

*P* values were calculated with the t test for quantitative variables or Chi-square test for categorical ones.

BMI, body-mass index; WHtR, waist-height rate; PBF, percentage of body fat; ALT, alanine aminotransferase; GLU, fasting glucose; TC, total cholesterol; TG, triglycerides; HDL-C, high-density lipoprotein cholesterol; LDL-C, low-density lipoprotein cholesterol; HOMA-IR, homeostasis model assessment of insulin resistance.

### DNA methylation differences between obese children and controls

Nine CpG sites (5 CpG units) detected in our study locates at the DNA region from 46801557 to 46801767 (GRCh37/hg19) (Table 2). Of these 9 CpG sites, previous EWAS studies have reported 3 CpG sites (46801557, 46801642 and 46801672) related with BMI of BMI change, whereas the remaining 6 CpG sites were not included in the Illumina 450K Bead Chip used by the two EWAS studies, nor had been reported to be associated with obesity-related phenotypes in other studies. We observed significantly higher methylation levels in obese children than in controls at both CpG5 and CpG11 (*P* <0.05) (Table 2).

**Table 2:** *HIF3A* DNA methylation values (in %) for each CpG unit in obese cases and controls.

CpG sites	Position	Obese Children (n = 107)	Controls (n = 105)	*P* Value
CpG1.2	46801557*,46801562	38.8(5.6, 25.5–56.5)	38.0(5.3, 27.0–53.5)	0.277
**CpG5**	46801642*	27.7(8.0, 12.5–56.5)	25.4(7.9, 9.0–53.0)	**0.044**
CpG6.7.8	46801669,46801672*,46801676	50.7(5.9, 37.0–69.0)	49.5(6.1, 30.0–64.0)	0.167
**CpG11**	46801699	48.3(9.3, 24.0–72.5)	45.4(9.7, 27.0–69.0)	**0.027**
CpG13.14	4,680,175,546,801,760	34.2(5.1, 20.5–45.0)	34.7(5.5, 25.0–56.0)	0.468

Data are mean (SD, range). *P* values are calculated with the t test. CpG sites with significant differences of methylation between groups are in bold (p<0.05).

*The 3 CpG sites reported by the previous EWAS study [9].

*HIF3A*, hypoxia inducible factor 3 alpha subunit; CpG, cytosine-phosphate-guanine dinucleotides.

### Associations between *HIF3A* methylation and metabolic phenotypes in all subjects

We used partial correlation analyses adjusted for age and gender, to examine the associations between *HIF3A* methylation levels and obesity-related anthropometric/metabolic phenotypes. As Table 3 shows, BMI, waist-height rate (WHtR), percentage of body fat (PBF), GLU, HOMA-IR, ALT were significantly associated with HIF3A methylation levels at CpG11 (r = 0.169~0.263, *P* <0.05). ALT was also associated with the methylation level at CpG5 (r = 0.199, *P* = 0.016), but with a relatively smaller correlation coefficient than that of CpG11. To control the effect of BMI on metabolic phenotypes, we then conducted partial correlation analyses adjusted for age, gender and BMI (LgBMI). Only the association between ALT and CpG11 remained to be significant (r = 0.226, *P* = 0.007), revealing that CpG11 may have a BMI-independent association with ALT (Table 4).

**Table 3:** Partial correlation between *HIF3A* methylation and metabolic indicators adjusted for age and gender.

	CpG1.2	CpG5	CpG6.7.8	CpG11	CpG13.14
BMI	0.041(0.580)	0.130(0.008)	0.065(0.379)	**0.173(0.019)**	-0.032(0.671)
WHtR	0.023(0.757)	0.109(0.141)	0.067(0.366)	**0.169(0.022)**	-0.035(0.635)
PBF	0.039(0.605)	0.128(0.088)	0.051(0.501)	**0.165(0.028)**	-0.045(0.554)
GLU	0.018(0.829)	0.088(0.287)	0.087(0.299)	**0.179(0.030)**	0.026(0.755)
TC	-0.112(0.176)	-0.030(0.718)	-0.041(0.618)	0.006(0.943)	-0.139(0.094)
LDL-C	-0.075(0.370)	0.040(0.630)	-0.008(0.920)	0.050(0.550)	-0.106(0.201)
HDL-C	-0.022(0.789)	-0.113(0.172)	-0.013(0.873)	-0.118(0.156)	0.002(0.978)
TG	0.038(0.646)	0.159(0.055)	0.061(0.464)	0.144(0.083)	-0.025(0.765)
HOMO-IR	0.031(0.713)	0.157(0.058)	0.120(0.149)	**0.186(0.024)**	-0.015(0.857)
ALT	0.080(0.337)	**0.199(0.016)**	0.149(0.072)	**0.263(0.001)**	0.096(0.247)

Values shown are partial correlation coefficients (P values) adjusted for age and gender. Coefficients with *P* values <0.05 are in bold.

Variables of BMI, WHtR and metabolic indicators have been Lg converted.

*HIF3A*, hypoxia inducible factor 3 alpha subunit; CpG, cytosine-phosphate-guanine dinucleotides. BMI, body-mass index; WHtR, waist-height rate; PBF, percentage of body fat; ALT, alanine aminotransferase; GLU, fasting glucose; TC, total cholesterol; TG, triglycerides; HDL-C, high-density lipoprotein cholesterol; LDL-C, low-density lipoprotein cholesterol; HOMA-IR, homeostasis model assessment of insulin resistance.

**Table 4:** Partial correlation between *HIF3A* methylation and metabolic indicators adjusted for age, gender and BMI.

	CpG1.2	CpG5	CpG6.7.8	CpG11	CpG13.14
WHtR	-0.093(0.273)	-0.097(0.254)	-0.028(0.740)	-0.019(0.825)	-0.091(0.286)
PBF	0.039(0.648)	0.010(0.904)	-0.031(0.714)	0.021(0.802)	0.023(0.787)
GLU	-0.010(0.906)	0.030(0.725)	0.052(0.540)	0.135(0.112)	0.030(0.724)
TC	-0.137(0.106)	-0.070(0.408)	-0.064(0.451)	-0.037(0.667)	-0.143(0.093)
LDL-C	-0.133(0.117)	-0.052(0.543)	-0.061(0.477)	-0.057(0.504)	-0.136(0.109)
HDL-C	0.032(0.711)	-0.005(0.956)	0.053(0.534)	0.023(0.790)	-0.013(0.883)
TG	0.007(0.934)	0.091(0.283)	0.018(0.829)	0.055(0.520)	-0.014(0.870)
HOMO-IR	-0.006(0.943)	0.075(0.379)	0.101(0.237)	0.090(0.288)	0.013(0.880)
ALT	0.061(0.471)	0.158(0.063)	0.133(0.117)	**0.226(0.007)**	0.131(0.122)

Values shown are partial correlation coefficients (P values) adjusted for age, gender and BMI. Coefficients with P values <0.05 are in bold.

Variables of BMI, WHtR and metabolic indicators have been Lg converted.

HIF3A, hypoxia inducible factor 3 alpha subunit; CpG, cytosine-phosphate-guanine dinucleotides. BMI, body-mass index; WHtR, waist-height rate; PBF, percentage of body fat; ALT, alanine aminotransferase; GLU, fasting glucose; TC, total cholesterol; TG, triglycerides; HDL-C, high-density lipoprotein cholesterol; LDL-C, low-density lipoprotein cholesterol; HOMA-IR, homeostasis model assessment of insulin resistance.

## Discussion

To the best of our knowledge, this study is the first study to validate the association between obesity and *HIF3A* DNA methylation in the population of Chinese children. Besides the original 3 CpG sites identified by the EWAS study [9], we have found a nearby CpG site (CpG11, 46801699) with a relatively stronger effect. Furthermore, we have reported a BMI-independent association between *HIF3A* methylation and ALT for the first time.

Two EWAS studies [9, 11] have uncovered that increased methylation at the HIF3A locus was associated with increased BMI or BMI change. Similarly, we found that obese children had a higher level of HIF3A methylation in the current study. The 3 CpG sites reported by Dick et al. [9] to be associated with BMI were all included in the 9 sites of our study, which were CpG1 (46801557, cg27146050 in EWAS), CpG5 (46801642, cg22891070), and CpG7 (46801672, cg16672562); the CpG site reported by Demerath et al. [11] to be associated with BMI change was also included in our study, which was CpG7 (46801672, cg16672562). However, it seems that CpG11, a CpG site 27bp downstream of CpG7 and not within the Illumina methylation chip, has a stronger effect than CpG1, CpG5 or CpG7 both in relation with obesity and with ALT.

The CpG sites tested in this study are all neighboring sites in intron 1 of the gene *HIF3A*. The new CpG site (46801699) locates at 142, 57 and 27 bp downstream the three previously identified CpG sites by EWAS [9]. *HIF3A* is a component of the hypoxia inducible transcription factor (HIF). HIF was first uncovered by Semenza et al. in 1992 during the research on erythropoietin gene. It composes a β subunit (HIF-β) and one of three α subunits (HIF-1α, HIF-2α, HIF-3α), and regulates responses to reduced oxygen concentrations on cellular and physiological levels [20]. *HIF3A*, the gene which encodes HIF-3α, locates at 19q13.2 with a length of 43kb and 19 introns, and has 8 possible kinds of alternate splicing [21]. HIF-3αhas not been investigated as thoroughly as the other α subunits, but it is usually regarded as a negative regulator of HIF-1αand HIF-2α[22].The causal relation between obesity and *HIF3A* methylation is far from definitive conclusions. The EWAS study [9] tended to suggest that the increased methylation level at the *HIF3A* locus may be a result of increased BMI, because they found that two nearby single nucleotide polymorphisms (SNPs, rs8102595 and rs3826795) were associated with *HIF3A* methylation but not associated with BMI. However, Huang et al. [23] showed that the HIF3A variant rs3826795 was associated with BMI changes through interactions with total or supplemental vitamin B2, vitamin B12 and folate, and further suggested that DNA methylation may casually affect body adiposity.

Other laboratory evidences have provided cues linking *HIF3A* and adiposity. ANAKA et al. [24] found that HIF-3αmRNA was induced during the adipose differentiation process, and ectopic expression of HIF-3αmRNA at the early stage of differentiation induced the expression of several kinds of adipocytes-related genes including Fabp4 and AMPKγ1. This implicates that *HIF3A* may act as a transcription regulator of some adipocytes-related genes. Heidbreder et al. [25] conducted a study in rats subjected to insulin-dependent hypoglycemia or insulin-independent cytoglucopenia provoked by 2-deoxy-D-glucose (2-DG), and suggested that the hypoglycemia or cytoglucopenia were fully effective to cause a rapid increase of HIF-3αmRNA levels, whereas other HIFs remained unaffected.

Plasma ALT is widely used to reflect the liver function [26]. It is shown that aminotransferase elevation is strongly associated with central adiposity and related features including dyslipidemia, diabetes and hypertension [27]. The link between BMI and ALT is possibly because obesity is involved in the visceral adipose deposition that causes hepatotoxic fatty acids [28], or because of the modulation by insulin resistance [29]. We haven’t found direct evidences linking *HIF3A* gene with plasma ALT or liver diseases, but some studies on *HIF1A*, hypoxia or liver diseases may give some cues. Tamaki et al. [30] detected increases in both ALT and HIF-1α protein levels in rats with liver cirrhosis. Stroka et al. [31] found that when exposed to hypoxia, liver HIF-1α protein reaches maximal levels after 1 h and gradually decreases to baseline levels after 4 h of continuous hypoxia, but HIF-1α mRNA levels had no significant changes. Lin et al. [32] showed that in Obstructive Sleep Apnea (OSA) subjects, plasma level of ALT was significantly increased with the aggravation of OSA, and oxygen desaturation index was the major contributing factor for elevated ALT. The function of *HIF3A* is far from clear. Recent findings of the EWAS studies and our study on *HIF3A* DNA methylation prompt that it is quite necessary to conduct further experimental studies about the function of *HIF3A*.

There are a few limitations to this study. Firstly, though we have found an independent association between *HIF3A* methylation and ALT, the case-control study design means that we cannot assess the causal relation directly from the results. Secondly, because of the lack of gene expression data, this study may not provide direct evidence of the expression of *HIF3A*. Thirdly, the ideal tissues to identify epigenetic variations related to obesity or ALT should be adipose issues or hepatic issues, but we only investigated the methylation levels in peripheral blood because of difficulties in obtaining tissues from children. However, multiple studies have used DNA methylation in peripheral blood [9, 11,33–34], and by analyzing the association between BMI and methylations sites in both blood and adipose tissue DNA, Dick et al. [9] supports the use of whole-blood DNA methylation profiling for identification of relevant epigenetic changes.

The sequence we tested have covered all the three CpG sites reported by previous EWAS study, which were CpG1 (46801557, cg27146050 in EWAS), CpG5 (46801642, cg22891070) and CpG7 (46801672, cg16672562). We have the exact methylation data of CpG5, but CpG1 can only be represented by CpG1.2 which is the average methylation level of CpG1 and CpG2, and so does CpG7 (represented by CpG6.7.8). This is because the MassARRAY method could not get the methylation level of each CpG sites separately when the CpG sites locate very closely, but give the average level of the several sites instead, which is one of the limitations of our study. So it is possible that the effects of CpG1 and CpG7 were diluted by nearby sites.

Of the three CpG sites previously reported to be associated with BMI, only CpG5 had significant association with obesity in the validation in this study. This can be also due to the relatively small sample size, because CpG1 and CpG7 showed a little higher methylation levels in obese children than controls though without statistical significance, which is in consistency with CpG5. As the CpG sites tested in the current study (including the new identified CpG site) locates at the intron, it’s hard to say that it can directly inhibit transcription by blocking transcription factor binding as promoter sequences. However, several studies focused on the intron epigenetic sites and suggested intron DNA methylation can indirectly prevent transcription [35, 36]. It still lacks evidence whether methylation of *HIF3A* CpG site affects *HIF3A* expression, and future studies should pay attention on the mechanism of *HIF3A* DNA methylation and its gene expression.

In summary, this study has validated the association between *HIF3A* methylation and obesity in Chinese children for the first time, reported a new CpG site at *HIF3A* associated with obesity, and explored a BMI-independent association between *HIF3A* methylation and plasma ALT level. The results not only provide new cues on the function of *HIF3A* methylation, but also provide evidence for identifying epigenetic factors of elivated ALT and may be useful for risk assessment and personalized medicine of liver diseases such as NAFLD.
